# SATURN MRI: study protocol for the statin use in intracerebral hemorrhage patients MRI ancillary study

**DOI:** 10.1186/s13063-025-09024-0

**Published:** 2025-08-30

**Authors:** Sarah Marchina, Lydia D. Foster, Sharon D. Yeatts, Pooja Khatri, Kimberlee Bernstein, Aaron Perlmutter, Elizabeth C. Heistand, Eric E. Smith, Mike Sharma, Joseph P. Broderick, Vasileios-Arsenios Lioutas, Joan Marti-Fabregas, Magdy Selim, Ashkan Shoamanesh

**Affiliations:** 1 Department of Neurology, Beth Israel Deaconess Medical Center, 330 Brookline Avenue, Boston, MA USA; 2 Department of Public Health Sciences, College of Medicine, Medical University of South Carolina, Charleston, SC USA; 3 McMaster University, Hamilton Health Sciences, Population Health Research Institute, Hamilton, ON Canada; 4 Department of Neurology, University of Cincinnati, Cincinnati, OH USA; 5 Department of Neurology, Hospital de La Santa Creu I Sant Pau, Barcelona, Spain; 6 Department of Clinical Neurosciences, University of Calgary, Calgary, AB Canada

**Keywords:** Statin, Intracerebral hemorrhage, Lobar, Cerebral small vessel disease, Outcome, MRI

## Abstract

**Background:**

The benefit-risk of statins in patients with lobar intracerebral hemorrhage (ICH) is under investigation in the StATins Use in intRacerebral hemorrhage patieNts (SATURN) trial. The relationship between statin use in ICH survivors, MRI markers of cerebral small vessel disease (CSVD), and outcomes such as recurrent ICH or major adverse cardiovascular or cerebrovascular events (MACCE) is unclear. The ancillary study, SATURN-MRI, intends to evaluate the interrelationship between statin use, the progression of MRI markers of CSVD, and cognitive and functional outcomes. Additionally, SATURN-MRI aims to assess whether baseline MRI markers of CSVD interact with statin continuation for the outcomes of recurrent ICH or MACCE in patients with lobar ICH.

**Methods:**

A target of 894 SATURN participants will undergo a baseline MRI within 7 days of randomization and a repeat MRI at the end of the 24-month follow-up period. Any SATURN subject without contraindication to MRI has the option to participate in SATURN MRI. MRIs will be reviewed by blinded central raters to assess for the presence and burden of markers of CSVD and their progression. The primary outcome is new cerebral microbleeds and/or sulci with cortical superficial siderosis identified on T2*-weighted (GRE, SWI or SWAN) images between baseline and end-study MRI. Additional outcomes include the progression of white matter hyperintensity and incidence of covert infarcts.

**Discussion:**

The results will provide insights about the interrelationship between the effects of statins, progression of MRI markers of CSVD, and functional and cognitive outcomes. They might lead to the validation of MRI markers as tools to assist with individualized decision-making regarding the effects of statins continuation/discontinuation in patients with lobar ICH.

**Trial registration:**

ClinicalTrials.gov NCT03936361. SATURN Trial was originally registered on May 1, 2019 and modified on February 28, 2022.

**Supplementary Information:**

The online version contains supplementary material available at 10.1186/s13063-025-09024-0.

## Introduction

A large percentage of spontaneous intracerebral hemorrhage (ICH) is caused by cerebral small vessel disease (CSVD) [1, 2], either hypertensive arteriopathy (arteriolosclerosis)—associated with non-lobar—or cerebral amyloid angiopathy (CAA), linked to lobar hemorrhages. There are several imaging markers of CSVD such as cerebral microbleeds (CMBs), cortical superficial siderosis (cSS), lacunes, white matter hyperintensities (WMH), and enlarged perivascular spaces (PVS) which can be identified using MRI. The presence and severity of CMBs and cSS in particular have been associated with an increased risk of first ICH and ICH recurrence in patients with CAA [3–5].

Patients with spontaneous lobar ICH often have varying degrees of underlying CSVD on MRI, and the effects of long-term statin therapy on the burden of CSVD have yet to be defined. There is evidence that statin use is independently associated with the presence and increased number of CMBs, especially in cortical locations [6–8] and that statin treatment can reduce incident covert brain infarcts [9]. Determining the association of statin therapy with accrual of CSVD is important, as the accumulation of microvascular insults potentiates vascular cognitive impairment, functional decline, and gait impairment in older age [10–12]. The presence and burden of CSVD markers, in particular CMBs and cSS, may additionally be useful as a screening tool to stratify the benefit-risk of statin therapy in ICH survivors taking statins and to monitor the effects of statins on the progression of these markers and potentially increased propensity to recurrent ICH. Many physicians take these imaging features into consideration to guide their therapeutic decisions in patients with ICH [13]. However, evidence from randomized controlled trials (RCTs) supporting the prognostic value of these imaging features in ICH patients is lacking.

The SATURN-MRI ancillary study provides a synergistic opportunity to examine the prognostic and pathophysiological characteristics of MRI biomarkers of CSVD within the scope of a large-scale, prospective RCT.

## Methods

### Study design

SATURN-MRI is an ancillary study of the parent StATins Use in intRacerebral hemorrhage patieNts (SATURN) trial which is an international, multi-center, pragmatic, prospective, randomized, open-label, Phase III clinical trial with blinded end-point assessment (PROBE) [14]. Briefly, SATURN aims to evaluate the risks of symptomatic recurrent ICH (i.e., any neurological deficits or clinical symptoms resulting in brain imaging and diagnosis of ICH) and major adverse cardiovascular or cerebrovascular events (MACCE) with continuation vs. discontinuation of statin drugs during a 2-year follow-up period in patients presenting with spontaneous lobar ICH while on a statin [14]. A subset of patients participating in SATURN will be enrolled in SATURN-MRI. Participation in SATURN-MRI is optional.

### Study objectives

#### Primary objective

To determine the effects of continued long-term statin therapy compared to statin discontinuation on the incidence of new hemorrhagic MRI markers (CMBs and cSS) on end-study MRI in patients with lobar ICH. We hypothesize that the proportion of patients with new CMBs and/or cSS on end-study MRI will be higher among those who continue statin therapy.

#### Secondary objectives

1. To assess whether the presence and burden of CMBs and cSS on baseline MRI modifies the effects of statin on the risk of recurrent symptomatic ICH and can potentially be used as an imaging marker to stratify the benefit-risk of statin therapy in patients with lobar ICH. We also aim to examine the modifying effects of these markers on the risk of MACCE.
2. To evaluate the effects of statin continuation vs. discontinuation on the incidence of new ischemic MRI markers (white matter hyperintensities (WMH) and covert infarcts) on end-study MRI.
3. To evaluate interactions between statin continuation vs. discontinuation, accrual of MRI markers of CSVD, and functional and cognitive outcomes at the end of the study at 24 months.

### Patient population

SATURN-MRI population will be drawn from the participants in the SATURN parent trial [14]. The inclusion/exclusion criteria for the SATURN trial have been previously described [14]. Briefly, patients aged 50 years or older presenting with a spontaneous lobar ICH while taking a statin drug who are randomized into SATURN will be asked to participate in SATURN MRI. Participants in SATURN MRI must meet all of the eligibility criteria for SATURN. In addition, [1] the subject or surrogate must opt in to participate in the MRI study; and [2] the subject must have no known contraindications to MRI such as claustrophobia, metal implants, or pacemaker. Participants will be prospectively enrolled at approximately 140 hospitals in the United States, Canada, and Spain.

### Randomization

Patients will be randomized into the SATURN trial using the following stratification variables: clinical site, statin dose and indication (primary vs. secondary prevention), current use or intent-to-use oral anticoagulants and/or antiplatelets in the long-term post-ICH, and severity of ICH upon presentation as assessed by baseline ICH volume. There is no additional randomization procedure for the SATURN-MRI ancillary study.

### Intervention

Participants will undergo a standardized brain MRI protocol during hospitalization within 7 days of randomization into SATURN and again within 30 days of the 24-month end-of-study period. Subjects who already undergo a baseline MRI as part of institutional practice are not required to undergo a repeat baseline study-specific MRI to participate in SATURN-MRI, provided that the MRI complies with the study imaging protocol (Table 1).

**Table 1:** SPIRIT figure

	**Study period**							
	Enrolment	Allocation	Post allocation					Close-out
TIMEPOINT	-t	0	T_1_	T_2_	T_3_	T_4_	T_8_	t_x_
ENROLMENT:								
Eligibility – screen	X							
Informed consent	X							
Randomization procedures								
Allocation		X						
INTERVENTIONS:								
Intervention A: Statin		X						
Intervention B: No Statin		X						
ASSESSMENT:								
MRI baseline	X							
MRI follow-up							X	

### MRI protocol

Study sites are required to use a standardized brain MRI protocol to minimize variability from site to site and keep the same field strength and T2*-weighted sequence for the same subject, whenever possible, for both the initial and follow-up MRI to maximize the precision of image analyses and incident lesions. The MRI protocol must include the following: T1-weighted, T2-weighted, fluid attenuated inversion recovery (FLAIR), diffusion-weighted, and T2*-weighted (either a gradient-echo [GRE], susceptibility-weighted [SWI], or T2*-weighted angiography [SWAN]) sequences with parameters consistent with Standards for Reporting Vascular Changes on Neuroimaging (STRIVE) consensus guidelines for imaging manifestations of small vessel disease [15] (Supplementary Table 1). MRI scanners with field strengths ranging from 1.5 to 3.0 T are allowed depending on the site’s capabilities.

### Imaging analyses

Experienced readers, blinded to clinical details including treatment assignment, at the imaging core lab at the Seaman Family MRI Center at the University of Calgary will analyze the images. FDA compliant OsiriX software (Pixmeo SARL, Geneva, Switzerland) will be used to view the images. All incident lesions will be defined according to consensus guidelines for CMBs detection and STRIVE [15]. CMBs will be categorized by number (0, 1–2, 3–10, or > 10) and location (lobar, deep, or mixed deep/lobar), while cSS will be categorized as focal (involving ≤ 3 sulci) or disseminated (if involving ≥ 4 sulci). Covert infarcts will be defined as lesions in a vascular territory that are hyperintense on T2-weighted or FLAIR and hypointense on T1-weighted images, and if subcortical, they need to be at least 3 mm in size and have a round or ovoid shape to distinguish them from a perivascular space. Infarcts will furthermore be classified by size (> 15 or ≤ 15 mm axial diameter) and location (including cortical vs. subcortical and by lobe). Dilated perivascular spaces (Virchow-Robin) will be distinguished from infarcts on the basis of location, morphology, diameter (generally < 3 mm), and the absence of gliosis. Diffusion-weighted (DWI) hyperintense lesions will be defined as remote areas outside of the perihematomal border that are hyperintense on DWI with either corresponding hypointensity or isointensity on acute diffusion coefficient (ADC) sequences. White matter hyperintensities (WMH) will be divided into periventricular or deep and rated visually using the Fazekas scale. Semi-automated quantitative measurement of WMH volume will also be carried out using a Lesion Extraction Tool (University of Calgary; Calgary, AB, Canada).

### Study outcomes

The primary outcome is new CMBs and/or sulci with cSS on T2*-weighted (GRE or SWI) images at end-study compared to baseline. Additional outcomes include the count of new CMBs, progression of WMH, and incidence of covert new non-lacunar infarcts between baseline and end-study MRIs. Clinical outcomes of (i) recurrent ICH, (ii) MACCE, (iii) cognitive decline (telephone T-Montreal Cognitive Assessment T-MoCA; https://mocacognition.com/), and (iv) poor functional outcome (modified Rankin Scale (mRS) 4–6) [16, 17] will also be assessed to determine if their relationship with statin use is modified by the presence and burden of CMBs and cSS on baseline MRI. The details on how functional and cognitive outcomes are assessed and collected are described elsewhere [14].

### Data and safety monitoring bodies

Both the SATURN trial and SATURN-MRI study are governed by the University of Cincinnati (UC) Institutional Review Board (IRB) which serves as the central IRB for sites in the United States. Individual Ethics Boards oversee the trial at participating Canadian and Spanish sites. The independent Data and Safety Monitoring Board (DSMB) is appointed by the US National Institute of Neurological Disorders and Stroke (NINDS) and is responsible for the oversight of the participants’ safety and advising NINDS regarding continuation/discontinuation of the trial. An independent Medical Safety Monitor is responsible for monitoring the study with regard to safety on an ongoing basis and communicating with the DSMB. The Data Coordination Unit at the Medical University of South Carolina (MUSC) is in charge of monitoring of the sites.

### Statistical considerations

#### Sample size calculations

The target sample size is 894, based on the following observations/assumptions. We previously reported that 57% of statin-treated patients admitted with acute lobar ICH had CMBs on MRI [6]. Although the estimated incidence rate of new CMBs per year in patients with lobar ICH on/off statins is unknown, the annual incidence rate in mixed stroke cohorts varies from 3 to 7% per year (average ~ 5% per year) [18–21]. In a cohort of 118 patients with probable CAA, 8.5% showed new CMBs without cSS progression, 18.6% showed cSS progression without new CMBs, and 9.3% showed new CMBs and cSS progression, for a total of 36.4% with progression of either CMBs and/or cSS over the 2.2 years of follow-up, or approximately 19% per year [18]. These data likely overestimate the incidence rate expected in SATURN-MRI because approximately 33% of SATURN patients are expected to have probable CAA, and another 40% possible CAA. Therefore, we opted to select a more conservative new CMB and/or cSS rate of 6% per year, for a total of 12% over 24 months in patients discontinuing statins. We postulated that statin therapy will increase the risk of new CMB and/or cSS at a rate that is similar to that reported for ICH in the SPARCL trial (HR 1.68, or absolute increase of 8% over 24 months). Based on these assumptions, a two-group comparison of independent proportions with 652 subjects will yield 80% power to detect an absolute increase of 8 percentage points associated with the continuation of statin therapy in SATURN. This sample size was further inflated to account for loss-to-follow-up (LTFU), withdrawal-of-consent, and death during the 2-year follow-up period of approximately 15%, as observed in other MRI sub-studies, such as NAVIGATE-ESUS [22] and PURE [23].

The sample size calculation assumes 1:1 statin continuation:discontinuation among subjects enrolled in the ancillary SATURN-MRI study, but this balance is not guaranteed. However, the power is maintained above 75% for allocation ratios in the interval between 0.6 (discontinuation arm 62.5% of the total sample) and 2.5 (discontinuation arm 28.6% of the total sample).

### Data analyses

The primary analysis will be conducted according to the intention-to-treat principle, meaning that all subjects are analyzed as a member of the treatment group to which they were randomly assigned in SATURN, even if the subject crosses over from one treatment arm to another. Secondary analyses will be conducted according to an on-assigned-treatment analysis. All models will be adjusted for the aforementioned randomization covariates as specified for SATURN (statin dose and indication, current use of or intent-to-use oral anticoagulants and/or antiplatelets post-ICH, and severity of enrolling ICH) [14].

#### Analysis related to the primary objective

We hypothesize that the proportion of patients with new CMBs and/or cSS on end-study MRI will be higher in the continuation arm. This hypothesis will be tested via a logistic regression model relating the presence of new lesions to treatment assignment.

#### Analyses related to the secondary aims

In order to explore whether the presence and burden (number) of CMBs on baseline MRI can be used as an imaging marker to stratify the risk/benefit of statin therapy, a proportional hazards model will be used to relate the time to symptomatic ICH recurrence to treatment assignment, the presence and burden of CMBs/cSS, and the corresponding interaction. The interaction term will be used to evaluate the heterogeneity of the treatment effect according to presence/burden, and the corresponding treatment effects and 95% confidence intervals will be constructed. The effects of statin continuation on the incidence of new ischemic MRI markers (WMH and covert infarcts) on end-study MRI will be evaluated via logistic regression model relating the presence of new markers to treatment assignment. Heterogeneity of the treatment effect across racial/ethnic subgroups will be explored by extending the proportional hazards model to include main effects for sex and racial/ethnic subgroups and their corresponding interactions with treatment and presence/number of new lesions.

The association between the incidence of new MRI markers on end-study MRI and deterioration of cognitive function, quantified by T-MoCA, and functional outcome, quantified by mRS, will be evaluated. As in the parent trial, the T-MoCA will be analyzed by considering cognitive decline (reduction of at least two points from the highest score to any subsequent assessment), as well as normal cognition (T-MoCA ≥ 20); the mRS will be dichotomized (0–3 vs 4–6). A generalized linear mixed effects model will be constructed for each outcome independently in order to relate outcome to treatment, time, and presence of new lesions; in addition to the adjustments for randomization covariates specified above, the models will include an indicator for baseline cognitive decline/dementia derived from the subject’s medical history.

### Lost-to-follow-up and missing data

If a participant in the ancillary MRI study has an ischemic stroke or recurrent ICH before the end of the 2-year follow-up period and a clinical MRI is obtained for stroke assessment at one of the participating sites, this MRI may serve as the end-of-study MRI if it complies with the study imaging protocol and the subject is unable to return for the 24-month follow-up MRI. If the clinical MRI indicates the presence of new markers, this indicator will be carried forward to the end-of-study timepoint. The data will be considered missing if the MRI does not show new markers and is performed more than 3 months prior to the participant’s anticipated end-of-study visit. Missing outcome data related to the incidence of MRI markers will be handled in the analysis via multiple imputation.

Multiple imputation is generally considered the least biased imputation method, since it incorporates uncertainty associated with the imputed value into the analysis. Briefly, logistic regression is used to derive a distribution for the primary outcome, using the primary analysis covariates (treatment, statin dose and indication, current use or intent-to-use oral anticoagulants and/or antiplatelets post-ICH, and severity of enrolling ICH) plus additional clinically relevant baseline variables. A random sample from the resulting distribution is used to impute values. Multiple sample data sets with complete primary outcome data are generated; each imputed data set will be analyzed according to the specified primary analysis, and the results from the multiple imputed data sets are combined to obtain a single set of parameter estimates. The multiple imputation method assumes missing at random (MAR) which means that the probability of missing outcome data can depend on the observed values of the individual but not on the missing values of the individual.

### Study organization and funding

The trial is funded within the framework of the US NIH StrokeNet by the NIH, NINDS, and NIA (UF1NS120871). The University of Cincinnati serves as the National Coordinating Center, and the Data Coordination Unit of the Department of Public Health Sciences within the College of Medicine at MUSC serves as the National Data Management Center. The overall responsibility and management of the trial lie with the prime site Beth Israel Deaconess Medical Center in Boston under the lead of the principal investigator (PI) Dr. Magdy Selim and Dr. Ashkan Shoamanesh (Multiple PI) at McMaster University in Canada.

## Discussion

Patients with spontaneous lobar ICH often have varying degrees of underlying CSVD on MRI, and the effects of long-term statin therapy on the burden of CSVD have yet to be defined. Statin therapy could interact with CSVD in various fashions. On one hand, statin use in ICH patients may be independently associated with the presence and number of CMBs, particularly in lobar locations [6, 8]. Statins possess plausible biological mechanisms by which they might increase the propensity for intracranial bleeding independent of lipid lowering by inhibiting platelets, decreasing thrombus formation, and enhancing fibrinolysis [24–26]. These properties of statins could accelerate the incidence/accrual of hemorrhagic markers of CSVD such as CMBs and cSS [7]. On the other hand, the putative anti-inflammatory, antiplatelet, endothelial stabilization, and cerebral vasoreactivity-enhancing properties of statins could reduce the accrual of covert infarcts and WMH over time [8, 27–31] Statins might additionally mitigate the contribution of concurrent atherothrombotic vascular disease to the accrual of covert infarcts and WMH [27–29, 31].

The SATURN trial is limiting eligibility to lobar ICH as a pragmatic means of deriving a CAA-enriched population. However, not all spontaneous lobar ICH are due to CAA in older populations, with a proportion resulting from underlying hypertensive arteriopathy [32]. The use of MRI would further help to improve the diagnosis of CAA through the use of the clinico-radiographic Boston Criteria. Moreover, even within CAA cohorts, response to stroke preventive treatments has been shown to differ depending on the severity of underlying disease manifested by MRI biomarkers [33]. MRI would allow assessment of treatment interactions between baseline MRI phenotypes suggestive of advanced CAA (which might be at greatest risk for increased recurrent ICH with continued statin therapy) and statin continuation vs. discontinuation. The potential to use MRI markers, such as CMBs and cSS, to identify patients who benefit or are harmed from long-term statin therapy could facilitate individualized decision-making and risk stratification and would be an important and novel contribution to the field which could have important cost- and resource-saving implications. In practice, the results of SATURN MRI will be interpreted in concert with those of the main SATURN trial. An increased risk of ICH recurrence with statin continuation in the SATURN main trial and as well an increased risk of ICH recurrence in patients with CMBs/cSS on baseline MRI in SATURN MRI would indicate higher absolute risks of ICH recurrence and lower numbers needed to harm in patients with CMBs/cSS. Conversely, if SATURN main results do not show an increased risk of ICH recurrence with statin continuation, but SATURN MRI shows increased risk in patients with CMBs/cSS, the results can similarly lead to individualization of treatment decisions according to MRI phenotypes. However, if both SATURN and SATURN MRI do not show increased risk of ICH recurrence with statin continuation, this would provide reassurance supporting the safety of statins among the highest risk subgroups of lobar ICH patients and could lead to avoidance of unnecessary neuroimaging in clinical practice and hence save costs.

Furthermore, observations from SATURN-MRI could lead to novel hypotheses for further research elucidating the pathogenesis of CSVD implicated in lobar ICH and ultimately lead to novel therapeutic targets aimed at mitigating the progression of microvascular cerebral injury and ensuing disability. The progression of markers of CSVD may be influenced by other variables such as adequacy of blood pressure (BP) control and use of anti-thrombotic drugs [30, 34]. The SATURN trial already adjusts for the use/intent-to-use of anti-thrombotic drugs in its randomization scheme. Participants are also asked to check and report their BP at follow-up assessments and are reminded at all points of contact of the importance of maintaining BP ≤ 130/80 mmHg. To address this, we also plan to perform post hoc correlation-type analyses to examine the impact of post-randomization variability in the use of anti-thrombotic drugs and adequacy of BP control on imaging outcomes.

## Summary and conclusions

SATURN-MRI provides a unique opportunity to evaluate the effects of long-term statin therapy on the burden of CSVD. As an ancillary study of the SATURN trial [14], SATURN-MRI will allow us to efficiently examine the prognostic and pathophysiological characteristics of MRI biomarkers of CSVD within the scope of a large-scale, prospective, randomized, controlled trial. The results from SATURN-MRI will have direct clinical relevance and could lead to validation of MRI imaging markers as tools to assist with individualized decision-making regarding the safety of statin use/continuation in ICH patients and the mitigation of late-life cognitive and functional decline.

## Supplementary Information

Additional file 1: Table S1.

Additional file 2: SPIRIT checklist.

## Data Availability

The final results of this study will be made available on the clinicaltrials.gov (NCT03936361), and data generated will be publicly available through the NINDS Archived Clinical Research Dataset. The study will follow NIH policies on data sharing (as described at http://grants2.nih.gov/grants/policy/data_sharing/data_sharing_guidance.htm) and any updates thereto. A complete de-identified dataset will be made available via the NIH Archived Clinical Research Datasets (https://www.ninds.nih.gov/Current-Research/Research-Funded-NINDS/Clinical-Research/Archived-Clinical-Research-Datasets) within one year of manuscript publication. The dataset will be made available for limited use to researchers whose proposed use of the data is approved by the NIH/NINDS in accordance with the terms and conditions of the NINDS data request form (https://www.ninds.nih.gov/sites/default/files/NINDS_Data_Request_Form_508C.pdf).

## Abbreviations

ICH: Intracerebral hemorrhage
CSVD: Cerebral small vessel disease
MACCE: Major adverse cardio- and cerebrovascular events
MRI: Magnetic resonance imaging
CAA: Cerebral amyloid angiopathy
CMB: Cerebral microbleed
cSS: Cortical superficial siderosis
WMH: White matter hyperintensities
PVS: Perivascular spaces
RCT: Randomized controlled trial
GRE: Gradient-echo
SWI: Susceptibility-weighted image
SWAN: Susceptibility-weighted angiography
UC: University of Cincinnati
IRB: Internal Review Board
NINDS: National Institute of Neurological Disorders and Stroke
NIH: National Institute of health
