# Supplementary material for: SATURN MRI: study protocol for the statin use in intracerebral hemorrhage patients MRI ancillary study
Source: Trials. 2025 Aug 30;26:323. doi: 10.1186/s13063-025-09024-0 (PMC12398981; doi:10.1186/s13063-025-09024-0)
Supplement: Supplementary file 1 — Additional file 1: Table S1. [file 13063_2025_9024_MOESM1_ESM.docx]

| **Sequence** | **T1** | **T2** | **FLAIR** | **DWI** | **GRE^+^** | **SWI/SWAN** |
| --- | --- | --- | --- | --- | --- | --- |
| **2D or 3D** | 2D or 3D | 2D | 2D | 2D | 2D | 2D or 3D |
| **Orientation** | Sagittal or coronal | Axial | Axial | Axial | Axial | Axial |
| **Thickness (mm)** | 2D: 3-5 mm  3D: 1 mm  (isotropic  Voxels) | 3-5 mm | 3-5 mm | 3-5 mm | 3-5 mm | 2D: 3-5 mm  3D: 1 mm |
| **In-plane resolution** | 1 x 1 mm | 1 x 1 mm | 1 x 1 mm | 2 x 2 mm | 1 x 1mm | 2D: 0.5 x 0.5 mm  (reconstructed) |
| **TR (msec)** | 2D: 450 - 600  3D: 2000 - 3000 | 3000-9000 | 7000 - 9000 | 3000 - 5000 | 300 - 800 | 30 - 50 |
| **TE (msec)** | 10 – 30 (min) | 80 - 120 | 100 - 150 (max) | 70 - 100 | 20 - 30 | 18 - 30 |
| **TI (msec)** | 800 - 1000 | - | ~2200 - 2500 | *b =* 1000 s/mm^2^ | Flip angle ~15-20 degrees | Flip angle ~1520 degrees |
| **NEX** | 1 | 1 | 1 | 1 - 3 | 1 | 1 |
| **FOV (cm)** | 24 - 26 | 24 - 26 | 24 - 26 | 24 - 26 | 24 - 26 | 24 - 26 |
| **Matrix** | 256 x 256 | 256 x 256 | 256 x 256 | 128 x 128 | 256 x 256 | 256 x 256 |
| **Gap** | 0 to 1mm | 0 to 1mm | 0 to 1mm | 0 to 1mm | 0 to 1mm | 0 to 1mm |

DWI, diffusion-weighted imaging; FLAIR, fluid-attenuated inversion recovery; GRE, gradient-recalled echo; NEX, number of excitations; SWI/SWAN, susceptibility-weighted imaging/susceptibility-weighted angiography; TE, echo time; TI, inversion time; TR, repetition time. Minimum resolution values are shown; higher resolution is acceptable.
